# Regulation of interpersonal distance in virtual reality: Implications for socio-emotional functioning in late adulthood

**DOI:** 10.1371/journal.pone.0323182

**Published:** 2025-05-08

**Authors:** Bozana Meinhardt-Injac, Isabelle Boutet, Laurence Chaby, Christoph von Castell, Robin Welsch

**Affiliations:** 1 Department of Developmental Psychology, Catholic University of Applied Sciences Berlin, Berlin, Germany; 2 School of Psychology, University of Ottawa, Ottawa, Canada; 3 Vision Action Cognition, Université Paris Cité, Paris, France; 4 Department of Psychology, Johannes Gutenberg-University Mainz, Mainz, Germany; 5 Department of Engineering Psychology, Aalto University, Espoo, Finland; New York University Abu Dhabi, UNITED ARAB EMIRATES

## Abstract

**Objective:**

Accurately interpreting emotional states from facial expressions is crucial for effective social interactions. This study investigates age-related differences in interpersonal distance (IPD) regulation and emotion recognition using a virtual reality (VR) environment. We examined how younger and older adults adjust their IPD in response to emotional expressions from virtual agents.

**Methods:**

Eighty participants, divided into older adults (OA) and younger adults (YA), took part in the study. Participants were immersed in a VR setup where they engaged in social interactions with happy or angry looking virtual agents. This behavioral task was complemented by a standardized emotion recognition task (ERT).

**Results:**

Results showed that both YA and OA preferred larger distances from angry-looking virtual agents compared to happy ones. No significant differences in IPD were found between the age groups. However, older adults were less accurate in recognizing facial expressions.

**Conclusion:**

These findings suggest that older adults can effectively regulate their social distance despite potential challenges in emotion recognition. The study underscores the importance of considering cognitive, perceptual, and motivational factors when examining the dynamics of emotional recognition and interpersonal distance in social contexts.

## Introduction

Forming an accurate representation of someone else’s emotional states from facial expression is fundamental for social interactions and survival [1] —for example, to retreat from a potentially threatening social interaction or to help someone in need. A growing body of research underscores the inherently social nature of emotions: they often emerge during interpersonal interactions, are regulated according to social norms and objectives, are expressed within social contexts, and significantly influence the behaviors and perceptions of others [2,3]. In addition, expressions shape behavior; they promote tendencies to approach or avoid. Specifically, distinct emotional expressions, such as anger or happiness, can elicit predictable, emotion-specific responses from observers, shaping their judgments and decision-making processes across various domains [4].

One effective approach to studying the impact of emotions on social interactions is to assess preferred interpersonal distance and the regulation of that distance in response to facial expressions. Interpersonal distance (IPD) is a physical distance that individuals choose to maintain between themselves and others during social interactions [5]. IPD acts as a nonverbal communicative mechanism and is dynamically adjusted based on the emotional cues perceived from interaction partners [6–9]. Virtual reality (VR) has emerged as a powerful tool for investigating how IPD is influenced by the social valence of stimuli [4,10,11]. For instance, Bönsch et al. [4] demonstrated that participants maintained larger distances from angry virtual agents compared to those displaying happiness, as well as from groups versus individuals. Additionally, Zibrek et al. [10] examined how perceived gender and the attractiveness of motion affect proximity in VR, revealing that attractive movements resulted in decreased proximity, while character gender did not have a significant effect. Iachini et al. [11] further clarified that both reachability and comfort distances are modulated by social factors, indicating a shared motor nature between these two dimensions.

However, research involving older adults remains limited and somewhat outdated. Studies examining IPD in late adulthood reveal complex patterns influenced by various factors. While some studies suggest that older adults maintain greater distances compared to younger individuals [12,13], evidence also indicates that IPD in old age may vary based on familiarity with the other person [14]. Specifically, a study by Mirlisenna et al [14] identifies two distinct developmental patterns in IPD: with strangers, there is a non-linear decrease from childhood to late adulthood, whereas with familiar individuals, a stepped decline occurs from pre-adolescence to adolescence, remaining stable until late adulthood. These discrepancies may arise from shifts in motivation as individuals age, specifically regarding the need for affiliation and personal feedback, and adherence to cultural norms [13]. Additionally, declines in physical and sensory capabilities associated with aging can significantly affect older adults’ use and control of personal space [15,16]. As older adults often experience changes in their perceptual systems, their ability to respond effectively to environmental stimuli may be compromised. Thus, older adults may struggle to establish an appropriate interpersonal distance, potentially leading to discomfort in social interactions [14].

Notably, healthy older adults aged 65 and above exhibit a marked decline in accuracy when recognizing facial expressions in computerized tasks [17–19]. This decline is particularly pronounced for negative emotions such as anger, fear, and sadness, while recognition of happiness remains relatively unaffected by aging. Recent studies cast doubt on this position and suggest that OAs’ cognitive and motivational resources are not fully utilized in typical computerized emotion recognition tasks. When contextual elements are incorporated—such as dynamic videos or multimodal facial-vocal cues—age-related deficits appear to diminish [20,21]. Enhancing the ecological validity of the stimuli may increase the personal relevance of the tasks, thereby fostering greater motivation and engagement among older participants [22,23]. Furthermore, research utilizing embodied conversational agents indicates that interactive environments can more effectively capture age-related variations in emotional perception [24,25].

Given the complex interplay of emotional recognition, interpersonal distance, and age, it is essential to investigate these dynamics within a more ecologically valid framework. This study aims to provide new insights into how different age groups manage social interactions and interpret emotions, highlighting the potential of VR to enhance our understanding of these processes. VR has gained recognition in psychological research for its capacity to enhance ecological validity and experimental control [26,27]. This technology is particularly relevant for exploring socio-emotional functions in older adults, where traditional methodologies often fail to capture the complexities of naturalistic settings. One of VR’s key advantages is its capacity to study social behavior under highly controlled conditions, allowing for consistent programming of virtual confederates to invade participants’ personal space in a standardized manner while maintaining external validity [28]. This balance between experimental control and ecological validity is crucial for advancing our understanding of social interactions in spatial contexts [28].

This study aims to address two research questions. Firstly, we examine how both young and older adults adjust their interpersonal distance (IPD) in response to a virtual agent expressing anger or happiness within an immersive VR environment. This methodological approach permits an analysis of the dynamic changes in IPD, providing insights into the influence of emotional expressions on social behavior. We hypothesize that both YAs and OAs will adjust their IPD based on the avatar’s facial expression [4]. Secondly, we examine the relationship between individual differences in IPD regulation and emotion recognition in both age groups, emphasizing how these groups adapt their social interactions in response to emotional cues from others. We hypothesize that IPD regulation will correlate with emotion recognition across both age groups, suggesting that OAs may face challenges not only in recognizing emotions but also in effectively regulating their interpersonal distance.

## Materials and methods

### Participants

Eighty (*N* = 80) participants took part in the experiment in return for partial course credit or monetary compensation. The sample was divided into two groups: older adults (OA) (*n* = 40, ♀ = 23, *M*_*age*_ = 67.03, *SD*_*age*_ = 4.20, Age range: 60–76 years) and younger adults (YA) (*n* = 40, ♀ = 23, *M*_*age*_* *=* *25.89; *SD*_*age *_= 3.52, Age Range = 18–34 years). Participants were recruited via advertisements on the campus of the Johannes-Gutenberg University Mainz, Germany and associated online communities. Older participants were involved in the University for Seniors at Johannes-Gutenberg University Mainz and none of them had cognitive problems as screened by the Mini Mental Status Test [29] (cutoff ≤ 24). Of the 40 OAs, 16 had a university degree, mostly in the natural sciences (i.e., math, physics, computer science, medicine). Thirty-three participants in the YA sample were enrolled in Bachelor’s (*N* = 11) or Master’s degree programs (*N* = 21). All participants were Caucasian.

Visual acuity was assessed using the Freiburg Visual Acuity Test – FrACT [30]. The average visual acuity (VA) score was.89 (SD = .30) in OA and 1.46 (SD = .27) in YA. The visual acuity was significantly higher in younger adults compared to older adults (*p* < .001). Therefore, the differences between YA and OA were analyzed by taking visual acuity (VA) measurements into account as a covariate (see below).

### Interpersonal distance in VR

#### Virtual agents.

**Fig 1:**
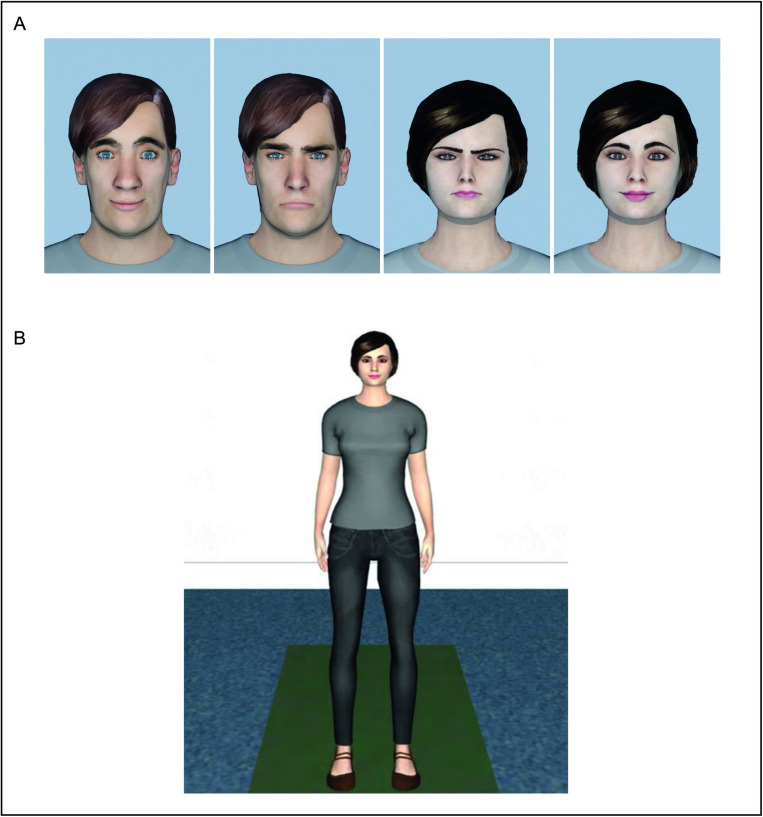
Virtual Agents. A) An example of the virtual agents with happy and angry facial expressions. B) An example of the full body of the virtual agents. Note that the body posture was neutral, and the size of the stimuli was adapted to the participant’s height.

Four different Caucasian virtual agents (two female and two male) were used to present a variety of social stimuli. The virtual agents were designed using Makehuman 1.1.0 Nightly Build, and their facial expressions were modulated in 3DSMAX in Autodesk to mirror Ekman pictures [31] (see Fig 1A). Research supports the notion that virtual agents can be used as proxies for real faces for emotion recognition [32]. The virtual agents were dressed uniformly in gray shirts and black pants (see Fig 1B). Agents were presented with either happy or angry facial expressions during the interactions. This standardized appearance and controlled variation in emotional expression were designed to isolate the effects of emotional expression on interpersonal distance (IPD) while minimizing other potential confounding variables.

### Apparatus and stimuli

**Fig 2:**
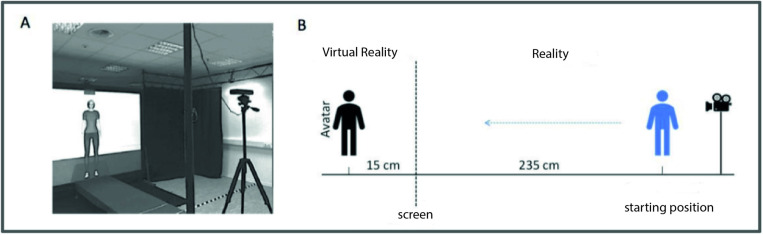
Experimental Setup A) VR environment. B) Procedure for measuring interpersonal distance (IPD).

Participants saw stereoscopic full-scale simulations on a large rear-projection screen (2.60 m wide × 1.95 m high). We used a 3D projector (projection design F10 AS3D) with a color resolution of 8 bits per channel, a display resolution of 1400 × 1050 (horizontal × vertical) pixels, and a refresh rate of 120Hz. Participants wore LCD shutter glasses (XPAND X102) synchronized via an infrared emitter, such that each eye received 60 frames per second. Participants’ individual inter-pupillary distance was measured by means of a pupil-distance meter and taken into account when computing the stereoscopic disparity of the VR environment. Measured from a distance of 2.35 m from the screen, the geometric field of view (FOV) was 58° horizontally and 45° vertically. The virtual FOV corresponded to the geometric FOV. The VR environment resembled the surrounding laboratory (see Fig 2A). The participants’ *movement* was tracked with a sampling frequency of 30 Hz using an infrared sensor (Microsoft Kinect®). The reference position was the participant’s spine. The accuracy of this method was previously validated in Hecht et al [33].

The virtual starting position of the characters was 15 cm behind the projection screen throughout all trials (see Fig 1B). To account for the influence of body height on IPD, the body height of the participant and the virtual agent were matched in all experiments by scaling the height of the virtual agents accordingly. To control for effects of gaze direction, the virtual agent`s eyes were dynamically adjusted so that they looked directly onto the observer’s bridge of the nose following the participant’s movement. Stimuli were presented using the VR software Vizard 5.

Participants were positioned standing on a platform in front of a large screen, facing the virtual agent. The initial interpersonal distance between the participant and the virtual agent was set at 250 cm, with the agent positioned 15 cm behind the projection screen and the participant standing 235 cm from it.

We manipulated two experimental factors within participants: virtual agent gender (two male, two female) and emotional expression (happy, angry). Each factor combination was repeated five times, resulting in 40 trials. Trials were presented in random order.

Prior to the main experiment, participants completed eight training trials with virtual agents showing neutral facial expressions, two trials for each virtual agent. The participants were instructed to walk towards the virtual agent until they reached a distance they considered comfortable for initiating a conversation with a stranger (e.g., asking for directions). Then, the participant confirmed the position and the IPD was logged.

After each trial, a black screen appeared, signaling participants to return to their starting positions. No time limit was given. Participants were instructed both in written and verbal form.

### Emotion recognition task (ERT)

The ERT is a computer-based forced-choice test where participants recognize the emotional facial expression of morphed faces [34]. It uses dynamic video clips, each showing a specific facial expression: anger, sadness, surprise, disgust, fear and happiness. The facial expression is morphed from neutral to full expression, at different levels of emotion intensity (0–40%, 0–60%, 0–80% und 0–100%). The number of frames and the video length ranges from 1–3 sec depending on the emotional intensity presented [34]. These video clips were shown for four individuals (two male, two female), which resulted in 96 trials. The ERT took about 12 minutes to complete.

#### Procedure.

The demographic questionnaires, FrACT, and emotion recognition test were administered prior to the VR experiment. All tests were conducted individually in a lab setting. An experienced experimenter was present at all times to provide technical support and instructions for the participants. Data collection took place between March 20, 2019, and March 14, 2022.

#### Ethics statement.

In accordance with the Declaration of Helsinki, all participants provided written informed consent prior to the study. Information about the study’s aims, methods, sources of funding, any possible conflicts of interest, and the institutional affiliations of the researchers was provided in written form, and participants were debriefed after the experiment. The procedure for the study was approved by the Ethics Committee of the Johannes-Gutenberg University Mainz, Germany.

### Statistical and power analysis

We aimed to investigate group differences in Interpersonal Distance (IPD) and emotion recognition, as well as the correlation between these variables. Given the significant age-related differences in visual acuity (see Participants section), we analyzed the data using repeated measures ANOVA to assess age and emotion-related differences, and ANCOVA to control for age-related differences in visual acuity.

Power calculations (using the power analysis module of Statistica 14.0.0.15, Tibco Soft Inc.) indicated that a sample size of N = 73 participants was sufficient to detect a standardized estimated population mean difference (OA < YA) of d = 0.80, with a power of 1- β = 0.9 for α = .05. This suggests that our sample size was adequate to identify between-group differences. However, a sample size of N = 100 is required to calculate a correlation coefficient for an expected population correlation of r = .20, and α = .05 with a power of.5. Therefore, correlations calculated with a sample size of N = 80 may not reliably detect expected effects.

Prior to conducting data analysis, we performed the Lilliefors test to assess the assumption of normality for the depended measures (IPD and emotion recognition). The results revealed no violation of normal distribution for IPD (Kolmogorov-Smirnov *d* = .07, *p* > .20; Lilliefors p > .20), IPD happy (Kolmogorov-Smirnov *d* = .06, *p* > .20; Lilliefors *p* > .20), IPD angry (Kolmogorov-Smirnov *d* = .06, *p* > .20; Lilliefors *p* > .20), nor for ERT mean score (Kolmogorov-Smirnov *d* = .08, *p* > .02; Lilliefors *p* > .20). However, there was violation from normal distribution for recognition of happy (Kolmogorov-Smirnov *d* = .23, *p* < .01; Lilliefors *p* < .01) and angry (Kolmogorov-Smirnov *d* = .15, *p* < .05; Lilliefors *p* < .01) emotional expression. These findings reflect celling effects reported elsewhere. The study was not pre-registered. All data are available at OSF: https://osf.io/cdsj3/?view_only=9dfd0a0e8a4f4334a722eb4b1dcba286

## Results

### Descriptive statistics and general effects

Descriptive statistics for all dependent measures, along with the results of a *t*-test comparing the performance of young and older adults on these measures, are shown in Table 1.

**Table 1:** Descriptive Statistics and Main effects of Age. IPD = interpersonal distance in meters (m); ERT = emotion recognition. The ERT score is calculated as the mean percentage correct (pc) across all six basic emotions.

	*Young adults*		*Older adults*		
	Mean	SD	Mean	SD	t-test (p)
IPD	1.30	.22	1.21	.29	.13
IPD happy	1.24	.18	1.16	.26	.16
IPD angry	1.37	.27	1.26	.34	.12
ERT	.68	.08	.56	.09	.001*
ERT happy	.96	.05	.91	.06	*.16*
ERT angry	.92	.10	.68	.19	.001*

### Age related differences in interpersonal distance (IPD) and IPD-regulation

**Fig 3:**
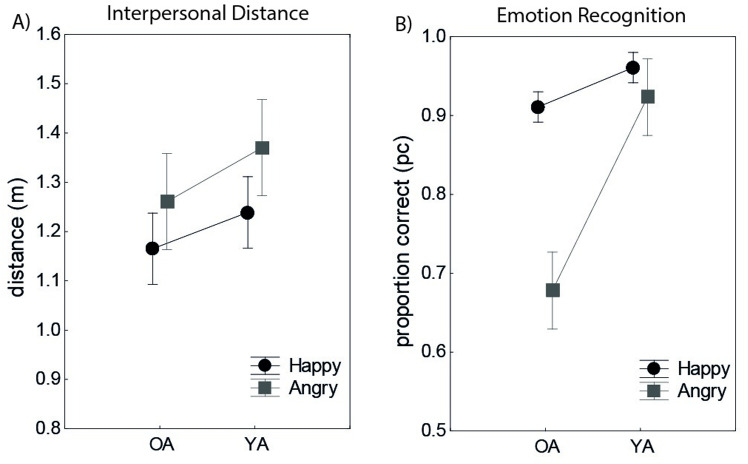
Results. A) No significant differences were found between young adults (YA) and older adults (OA) in interpersonal distance (IPD) or in its regulation based on the emotional expression of the virtual agent in virtual reality. B) Older adults were less accurate than young adults in recognizing angry (*η*^2^ = .39) and happy (*η*^2^ = .14) emotional expressions in a standardized face emotion recognition test (ERT).

We computed a rmANOVA on the individual mean IPDs (pooled across all virtual agents for every participant and facial expression) with Age group as a between-subjects factor and Facial expression as a within-subjects factor. Participants preferred larger distances towards angry-looking as compared to happy-looking virtual agents (*F*(1, 78) = 50.5, p < .001, *η*2 = .39). The main effect of Age group, *F*(1, 78)  = 2.41, p = .13, *η*2 = .03, was not significant, nor was the Age group x Facial expression interaction *F*(1, 78) = 1.24, p = .26, *η*2 = .01. Thus, the effect of facial expression remained unaffected by Age group (Fig 3A).

Next, we conducted an analysis of covariance (ANCOVA) with mean IPD as the dependent measure, Age group as the between-subject factor, and Visual acuity (VA) as the covariate. The results showed no significant differences in IPD between YA and OA (*F*(1,77) = 1.46, *p* = .22) and no significant effect of VA on IPD (*F*(1,77) = .002, *p* = .88). This was also confirmed in an ANCOVA with IPD to happy and angry faces as the dependent measures (all *p* > .10).

### Age related differences in Emotion Recognition (ER)

**Fig 4:**
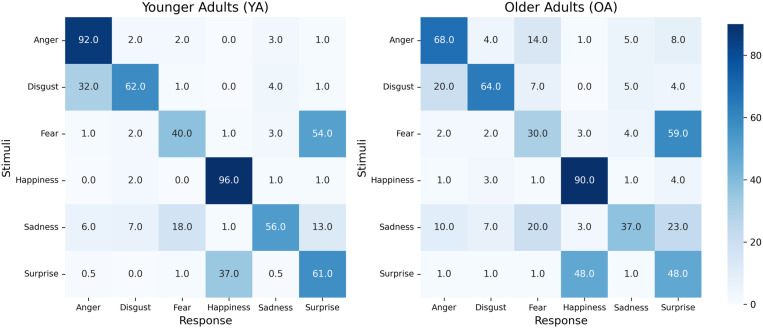
Emotion Confusion. In YA, anger was recognized accurately and was barely confused with other emotions. In OA, angry faces were categorized as fearful in 14% of all responses. Happiness was accurately recognized in both age groups. Very rare confusions occurred with disgust in YA and with disgust and surprise in OA.

We calculated a repeated-measures analysis of variance (rmANOVA) on the percentage of correct (pc) responses with Facial expression (6; Anger, Disgust, Fear, Happiness, Sadness, Surprise) as a within-subjects factor and Age group as a between-subjects factor. The main effects of Age group, *F*(1, 78) = 35.67, *p* < .001, *η*2 = .31 and Facial expression, *F*(5,390) = 152.49, *p* < .001, *η*2 = .66, were significant. These main effects were qualified by an Age group x Facial expression interaction, *F*(5, 390) = 7.16, *p* < .001, *η*2 = .08. Post-hoc t-tests revealed significant differences in recognition of Anger (*t*(1,78)=50,26, *p* < .001), Fear (*t*(1,78)=7.13, *p* < .01, Happiness (*t*(1,78)=13.28, *p* < .001, Sadness (*t*(1,78)=19.39, *p* < .001) and Surprise (*t*(1,78)=11.95, *p *< .001), bu*t* not in recogni*t*ion of Disgust (*t*(1,78)=.20, *p* = .64). The percent of correct responses as well as the error types in YA and OA are shown in Fig 4.

Next, we conducted an analysis of covariance (ANCOVA) with mean percentage (pc) of emotion recognition across all emotions, and pc for happy and angry faces as the dependent variables, Age group as the between-subject factor, and Visual acuity (VA) as the covariate. Overall, emotion recognition was more accurate in young adults (YA) than in OA (*F*(1,77)=23.1, *p* < .001, η2 = .23), and there was no significant effect of VA on emotion recognition (*F*(1,77) =.066, *p *= .41, η2 = .008. However, recognition of anger was marginally affected by VA (*F*(1,77)=3.5, *p* = .06, η2 = .04), while this was not the case for happy faces (*F*(1,77)=.04, *p* = .83, η2 = .0).

### Correlation between IPD and ER

Next, we conducted a correlation analysis to examine the relationship between emotion recognition, recognition of angry and happy facial expressions, and interpersonal distance to happy and angry faces (see Table 2). However, none of the correlations proved to be statistically significant (all *p* > .10), suggesting no relationship between emotion recognition and the regulation of interpersonal distance to emotional virtual agents.

**Table 2:** Correlation matrices. correlations among interpersonal distance (IPD) and emotion recognition test (ERT) measures. the analysis was conducted with a sample size OF *N* = 80.

	IPD	IPD happy	IPD Angry
ERT	*r(80)*=.01, *p* = .93	*r(80)=*-.04; *p* = .70	*r(80)*=.05; *p* = .67
ERT happy	*r(80)*=.02; *p* = .88	*r(80)*=-.002; *p* = .99	*r(80)=*03; *p* = .79
ERT angry	*r(80)*=.17; *p* = .13	*r(80)*=.16; *p* = .14	*r(80)*=.17; *p* = .12

## Discussion

In the present study, we investigated age-related differences in interpersonal distance (IPD) regulation and emotion recognition using a virtual reality (VR) environment. Our findings revealed that both younger and older adults adjusted their IPD based on the emotional expressions of virtual agents, preferring larger distances from angry-looking virtual agents compared to happy-looking ones. However, there were no significant differences in IPD between the age groups. Additionally, older adults demonstrated lower accuracy in recognizing angry facial expressions compared to younger adults, while only marginal age-related differences were found for happy expressions. These results align with previous research indicating that older adults experience challenges in recognizing negative emotions such as anger, while their ability to recognize positive emotions like happiness remains relatively intact [17–19]. The lack of significant age-related differences in IPD suggests that older adults can effectively regulate their social space in a manner similar to younger adults, despite potential deficits in emotion recognition. Furthermore, the results align with recent studies indicating a relative stability in IPD throughout the adult lifespan [14]. In the following sections, we will discuss several possible explanations for our findings.

### Cognitive and Perceptual Decline.

The observed deficits in recognizing expressions among older adults could be attributed to age-related declines in cognitive and perceptual abilities [17,20,35,36]. One possible explanation is that OA’s cognitive resources are not utilized in relatively artificial laboratory tasks such is the ERT [37]. Note that, when social vigilance and richness of social information is high, as in our virtual environment, differences between age-groups in social behavior vanish [38]. Furthermore, VR provides high social and sensory immersion and may particularly benefit older adults by enhancing engagement and emotional responses [24]. Alternatively, accordingly to perceptual degradation hypothesis, older adults’ ability to perceive and process facial cues relevant for emotion detection might be negatively affected by the loss of visual acuity [35]. Our study provides some evidence, albeit not strong, for this hypothesis. Visual acuity was positively associated with difficulties in recognizing angry but not happy facial expressions. This finding adds to a body of evidence suggesting that age deficits in low-level vision can negatively impact cognitive and perceptual processes [39–41].

### Compensatory Mechanisms.

The fact that older adults adjusted their interpersonal distance [IPD] appropriately may suggest that compensatory mechanisms, such as increased reliance on contextual cues or prior social experience, may be at play. Research has shown that social-cognitive skills improve through midlife and often continue to advance into later adulthood. For instance, older adults tend to reason more effectively about everyday interpersonal problems [42] and use a greater variety of problem-solving strategies, demonstrating greater sensitivity to situational contexts [43]. These strategies are often adaptive and reflect the specific experiences and life circumstances of middle-aged and older adults [44]. Moreover, automatic threat detection remains intact in older age [45]. In contrast to younger adults, older adults are generally more effective at navigating their social environment, likely due to years of accumulated social experience [37,44]. Thus, it is important to interpret deficits among age groups in terms of practical relevance. An age-related loss of 10–15% accuracy in emotion recognition may not overwhelm expertise in social interaction behavior acquired over a lifetime, which is consistent with our findings of IPD in virtual reality (VR). However, it is important to note that our study does not offer direct evidence regarding how these compensatory mechanisms may influence the regulation of IPD in VR.

### Social Isolation and Loneliness.

The physical distancing requirements imposed by COVID-19 led to an increased preferred interpersonal distance (IPD), a trend that may continue even after the pandemic [46]. During this period, the global preferred IPD increased by 54%, with more pronounced increases observed among individuals who are particularly vulnerable to disease [47]. Furthermore, chronic loneliness, which is reported more frequently among older adults, is linked to a greater interpersonal distance [48]. Consequently, we cannot exclude the possibility that the findings of the present study reflect differences in COVID-19 distancing behaviors between younger and older adults, as well as potential long-term effects. However, the impact of COVID-19 on interpersonal distance is multifaceted. For instance, Cartaud et al. [49] found that IPD decreased when interacting with virtual agents wearing face masks, likely due to an increase in perceived trustworthiness. Thus, it remains an open question how social isolation and loneliness may have influenced interpersonal distance (IPD) and obscured potential differences in IPD between younger and older adults.

### Emotion Confusion.

Emotion confusion can be conceptually defined as an individual’s tendency to misinterpret one facial expression as another (e.g., perceiving a happy face as sad) [50]. Error patterns in facial emotion recognition may reveal distinct deficits in emotion recognition that contribute to these misinterpretations. Confusion matrices, typically relevant in neurodiverse samples such as individuals with schizophrenia and dementia, can help determine whether specific error patterns exist across different groups [50,51]. In these studies errors were predominantly observed in response to negative emotions. These incorrect responses primarily occurred between emotions within the same valence dimension (e.g., fear vs. anger) and seemed to involve subtle discrimination errors, rather than between different valence dimensions (e.g., happiness vs. sadness) [52]. Consequently, errors primarily occur with emotions that are perceptually similar. Focusing on error profiles, our findings demonstrate that the types of errors made regarding target emotions are similar across age groups. However, older adults are more likely to confuse anger with fear, while young adults rarely mix anger with other emotions. Both age groups consistently differentiate between anger and happiness. The capacity of both older and younger adults to accurately differentiate between positive and negative valence in emotional stimuli [53] may serve as a potential explanation for the absence of age effects observed in the IPD measure while age effects were observed in the emotion recognition measure. OA may have chosen larger IPD for angry vs. happy avatars despite confusing anger and fear because both of these emotions are negative and signal threat.

### Limitations

When discussing virtual reality (VR), many people typically picture an immersive and interactive experience involving headsets and various interactive devices. In this study, however, the equipment and environment were centered around a 3D projector, which may differ from conventional VR setups. Still, the application of VR adds a level of ecological validity and control that is valuable.

Additionally, the Interpersonal Distance (IPD) task and the Emotion Recognition Test (ERT) used different types of stimuli. The ERT focused on real human faces to evaluate dynamic facial emotion recognition, while the IPD task utilized computer-generated (static) faces. This variation in stimuli might contribute to the lack of correlation observed between the two tasks, as well as our relatively small sample size.

## Conclusions

In conclusion, our study demonstrates that older adults, despite facing challenges in recognizing negative emotions, can effectively regulate their interpersonal distance in social interactions in response to the emotional expression of others. Moreover, while social behavior in VR is inherently artificial and may not necessarily correspond to real-life behavior, research suggests that IPD effects typically do not differ between real and virtual environments [33,54]. This underscores the utility of VR as a tool for studying social interactions in a controlled yet ecologically valid manner. Our findings also emphasize the importance of considering various factors—cognitive, perceptual, and motivational—in future studies examining the relationship between emotion recognition and social functioning in older adults.

## References

[1] Bach DR, Dayan P. Algorithms for survival: a comparative perspective on emotions. Nat Rev Neurosci. 2017;18(5):311–9. doi: 10.1038/nrn.2017.35 28360419

[2] van Kleef GA, Côté S. The social effects of emotions. Annu Rev Psychol. 2022;73:629–58. doi: 10.1146/annurev-psych-020821-010855 34280326

[3] Tracy J. L., Randles D., Steckler C. M. The nonverbal communication of emotions. Curr. Opin. Behav. Sci. 2015;3:25–30.

[4] Bonsch A, Radke S, Overath H, Asche LM, Wendt J, Vierjahn T, et al. Social VR: how personal space is affected by virtual agents’ emotions. 2018 IEEE Conference on Virtual Reality and 3D User Interfaces (VR). Reutlingen: IEEE; 2018. p. 199–206. doi: 10.1109/vr.2018.8446480

[5] Hayduk L. A. Personal space: where we now stand. Psychol. Bull. 1983;94:293–335.

[6] Cartaud A, Ruggiero G, Ott L, Iachini T, Coello Y. Physiological response to facial expressions in peripersonal space determines interpersonal distance in a social interaction context. Front Psychol. 2018;9:657. doi: 10.3389/fpsyg.2018.00657 29867639 PMC5949865

[7] Marsh AA, Ambady N, Kleck RE. The effects of fear and anger facial expressions on approach- and avoidance-related behaviors. Emotion. 2005;5(1):119–24. doi: 10.1037/1528-3542.5.1.119 15755225

[8] Welsch R, Hecht H, Von Castell C. Psychopathy and the regulation of interpersonal distance. Clin. Psychol. Sci. 2018;6:835–47.

[9] Lebert A, Vergilino-Perez D, Chaby L. Keeping distance or getting closer: How others’ emotions shape approach-avoidance postural behaviors and preferred interpersonal distance. PLoS One. 2024;19(2):e0298069. doi: 10.1371/journal.pone.0298069 38306322 PMC10836711

[10] Zibrek K, Niay B, Olivier A-H, Pettre J, Hoyet L, McDonnell R. Proximity in VR: The Importance of Character Attractiveness and Participant Gender. 2022 IEEE Conference on Virtual Reality and 3D User Interfaces Abstracts and Workshops (VRW). Christchurch, New Zealand: IEEE; 2022. p. 672–3. doi: 10.1109/vrw55335.2022.00187

[11] Iachini T, Coello Y, Frassinetti F, Ruggiero G. Body space in social interactions: a comparison of reaching and comfort distance in immersive virtual reality. PLoS One. 2014;9(11):e111511. doi: 10.1371/journal.pone.0111511 25405344 PMC4236010

[12] Pochwatko G. Karpowicz B. Chrzanowska A. Kopeć W. Interpersonal distance in VR: reactions of older adults to the presence of a virtual agent. 2021. Preprint at http://arxiv.org/abs/2101.01652

[13] Winogrond IR. A comparison of interpersonal distancing behavior in young and elderly adults. Int J Aging Hum Dev. 1981;13(1):53–60. doi: 10.2190/TT33-D5JK-N23N-6YMT 7345031

[14] Mirlisenna I, Bonino G, Mazza A, Capiotto F, Cappi GR, Cariola M, et al. How interpersonal distance varies throughout the lifespan. Sci Rep. 2024;14(1):25439. doi: 10.1038/s41598-024-74532-z 39455677 PMC11511920

[15] Remland MS, Jones TS, Brinkman H. Interpersonal distance, body orientation, and touch: effects of culture, gender, and age. J. Soc. Psychol. 1995;135:281–97. 7650932 10.1080/00224545.1995.9713958

[16] Webb JD, Weber MJ. Influence of sensory abilities on the interpersonal distance of the elderly. Environ. Behav. 2003;35:695–711.

[17] Ruffman T, Henry JD, Livingstone V, Phillips LH. A meta-analytic review of emotion recognition and aging: Implications for neuropsychological models of aging. Neurosci. Biobehav. Rev. 2008;32:863–881. 18276008 10.1016/j.neubiorev.2008.01.001

[18] Hayes GS, McLennan SN, Henry JD, Phillips LH, Terrett G, Rendell PG, et al. Task characteristics influence facial emotion recognition age-effects: A meta-analytic review. Psychol Aging. 2020;35(2):295–315. doi: 10.1037/pag0000441 31999152

[19] Gonçalves AR, Fernandes C, Pasion R, Ferreira-Santos F, Barbosa F, Marques-Teixeira J. Effects of age on the identification of emotions in facial expressions: a meta-analysis. PeerJ. 2018;6:e5278. doi: 10.7717/peerj.5278 30065878 PMC6064197

[20] Grainger SA, Henry JD. Absence of age differences in emotion perception and gaze patterns using a contextually rich film-based assessment. Q J Exp Psychol (Hove). 2023;76(9):2017–27. doi: 10.1177/17470218221141644 36376992

[21] Chaby L, Boullay VL, Chetouani M, Plaza M. Compensating for age limits through emotional crossmodal integration. Front Psychol. 2015;6:691. doi: 10.3389/fpsyg.2015.00691 26074845 PMC4445247

[22] Carstensen LL, Isaacowitz DM, Charles ST. Taking time seriously: A theory of socioemotional selectivity. Am. Psychol. 1999;54:165–81. 10199217 10.1037//0003-066x.54.3.165

[23] Isaacowitz DM, Freund AM, Mayr U, Rothermund K, Tobler PN. Age-related changes in the role of social motivation: implications for healthy aging. J Gerontol B Psychol Sci Soc Sci. 2021;76(Suppl 2):S115–24. doi: 10.1093/geronb/gbab032 33881524

[24] Pavic K, Chaby L, Gricourt T, Vergilino-Perez D. Feeling virtually present makes me happier: the influence of immersion, sense of presence, and video contents on positive emotion induction. Cyberpsychol Behav Soc Netw. 2023;26(4):238–45. doi: 10.1089/cyber.2022.0245 37001171 PMC10125398

[25] Pavic K, Oker A, Chetouani M, Chaby L. Age-related changes in gaze behaviour during social interaction: An eye-tracking study with an embodied conversational agent. Q J Exp Psychol (Hove). 2021;74(6):1128–39. doi: 10.1177/1747021820982165 33283649

[26] Vasser M, Aru J. Guidelines for immersive virtual reality in psychological research. Curr Opin Psychol. 2020;36:71–6. doi: 10.1016/j.copsyc.2020.04.010 32563049

[27] Pan X, Hamilton AF de C. Why and how to use virtual reality to study human social interaction: The challenges of exploring a new research landscape. Br J Psychol. 2018;109(3):395–417. doi: 10.1111/bjop.12290 29504117 PMC6055846

[28] Blascovich J, Loomis J, Beall AC, Swinth KR, Hoyt CL, Bailenson JN. Target article: immersive virtual environment technology as a methodological tool for social psychology. Psychological Inquiry. 2002;13(2):103–24. doi: 10.1207/s15327965pli1302_01

[29] Folstein MF, Robins LN, Helzer JE. The mini-mental state examination. Arch Gen Psychiatry. 1983;40(7):812. doi: 10.1001/archpsyc.1983.01790060110016 6860082

[30] Bach M. The freiburg visual acuity test—automatic measurement of visual acuity. 1996. 10.1097/00006324-199601000-00008 8867682

[31] Ekman P, Friesen WV. Facial action coding system. PsycTESTS Dataset. 2019. doi: 10.1037/t27734-000

[32] Joyal CC, Jacob L, Cigna M-H, Guay J-P, Renaud P. Virtual faces expressing emotions: an initial concomitant and construct validity study. Front Hum Neurosci. 2014;8:787. doi: 10.3389/fnhum.2014.00787 25324768 PMC4179743

[33] Hecht H, Welsch R, Viehoff J, Longo MR. The shape of personal space. Acta Psychol (Amst). 2019;193:113–22. doi: 10.1016/j.actpsy.2018.12.009 30622020

[34] Kessels RPC, Montagne B, Hendriks AW, Perrett DI, de Haan EHF. Assessment of perception of morphed facial expressions using the Emotion Recognition Task: normative data from healthy participants aged 8-75. J Neuropsychol. 2014;8(1):75–93. doi: 10.1111/jnp.12009 23409767

[35] Boutet I, Meinhardt-Injac B. Age differences in face processing: the role of perceptual degradation and holistic processing. J Gerontol B Psychol Sci Soc Sci. 2019;74(6):933–42. doi: 10.1093/geronb/gbx172 29373754

[36] Prete G, Ceccato I, Bartolini E, Di Crosta A, La Malva P, Palumbo R, et al. Detecting implicit and explicit facial emotions at different ages. Eur J Ageing. 2024;21(1):8. doi: 10.1007/s10433-024-00805-1 38499844 PMC10948669

[37] Ziaei M, Fischer H. Emotion and aging. In: Neuroimaging Personality, Social Cognition, and Character. Elsevier; 2016. p. 259–78. doi: 10.1016/b978-0-12-800935-2.00013-0

[38] Stanley JT, Isaacowitz DM. Caring more and knowing more reduces age-related differences in emotion perception. Psychol Aging. 2015;30(2):383–95. doi: 10.1037/pag0000028 26030775 PMC4451607

[39] Lindenberger U, Ghisletta P. Cognitive and sensory declines in old age: gauging the evidence for a common cause. Psychol Aging. 2009;24(1):1–16. doi: 10.1037/a0014986 19290733

[40] Monge ZA, Madden DJ. Linking cognitive and visual perceptual decline in healthy aging: The information degradation hypothesis. Neurosci. Biobehav. Rev. 2016;69:166–73. 27484869 10.1016/j.neubiorev.2016.07.031 PMC5030166

[41] Boutet I, Dawod K, Chiasson F, Brown O, Collin C. Perceptual similarity can drive age-related elevation of false recognition. Front Psychol. 2019;10:743. doi: 10.3389/fpsyg.2019.00743 31143137 PMC6520656

[42] Cornelius SW, Caspi A. Everyday problem solving in adulthood and old age. Psychol Aging. 1987;2(2):144–53. doi: 10.1037//0882-7974.2.2.144 3268204

[43] Blanchard-Fields F, Jahnke HC, Camp C. Age differences in problem-solving style: the role of emotional salience. Psychol Aging. 1995;10(2):173–80. doi: 10.1037//0882-7974.10.2.173 7662177

[44] Hess TM, Osowski NL, Leclerc CM. Age and experience influences on the complexity of social inferences. Psychol Aging. 2005;20(3):447–59. doi: 10.1037/0882-7974.20.3.447 16248704

[45] Mather M, Knight MR. Angry faces get noticed quickly: threat detection is not impaired among older adults. J Gerontol B Psychol Sci Soc Sci. 2006;61(1):P54-7. doi: 10.1093/geronb/61.1.p54 16399942

[46] Welsch R, Wessels M, Bernhard C, Thönes S, von Castell C. Physical distancing and the perception of interpersonal distance in the COVID-19 crisis. Sci Rep. 2021;11(1):11485. doi: 10.1038/s41598-021-90714-5 34075094 PMC8169674

[47] Croy I, Heller C, Akello G, Anjum A, Atama C, Avsec A, et al. COVID-19 and social distancing: a cross-cultural study of interpersonal distance preferences and touch behaviors before and during the pandemic. Crosscult. Res. 2023;58(1):41–69. doi: 10.1177/10693971231174935

[48] Saporta N, Scheele D, Lieberz J, Stuhr-Wulff F, Hurlemann R, Shamay-Tsoory SG. Opposing association of situational and chronic loneliness with interpersonal distance. Brain Sci. 2021;11(9):1135. doi: 10.3390/brainsci11091135 34573157 PMC8471414

[49] Cartaud A, Quesque F, Coello Y. Wearing a face mask against Covid-19 results in a reduction of social distancing. PLoS One. 2020;15(12):e0243023. doi: 10.1371/journal.pone.0243023 33284812 PMC7721169

[50] Lee S-C, Lin G-H, Shih C-L, Chen K-W, Liu C-C, Kuo C-J, et al. Error patterns of facial emotion recognition in patients with schizophrenia. J Affect Disord. 2022;300:441–8. doi: 10.1016/j.jad.2021.12.130 34979185

[51] Wang Y, Zhu Z, Chen B, Fang F. Perceptual learning and recognition confusion reveal the underlying relationships among the six basic emotions. Cogn Emot. 2019;33(4):754–67. doi: 10.1080/02699931.2018.1491831 29962270

[52] Gressie K, Kumfor F, Teng H, Foxe D, Devenney E, Ahmed RM, et al. Error profiles of facial emotion recognition in frontotemporal dementia and Alzheimer’s disease. Int Psychogeriatr. 2024;36(6):455–64. doi: 10.1017/S1041610223000297 37039500

[53] Meinhardt-Injac B, Altvater-Mackensen N, Mohs A, Goulet-Pelletier J-C, Boutet I. Emotion processing in late adulthood: the effect of emotional valence and face age on behavior and scanning patterns. Behav Sci (Basel). 2025;15(3):302. doi: 10.3390/bs15030302 40150197 PMC11939290

[54] Iachini T et al. Peripersonal and interpersonal space in virtual and real environments: effects of gender and age. J. Environ. Psychol. 2016;45:154–64.

